# Community-based aftercare following an emergency department presentation for attempted suicide or high risk for suicide: study protocol for a non-randomised controlled trial

**DOI:** 10.1186/s12889-019-7751-8

**Published:** 2019-10-26

**Authors:** Vida V. Bliokas, Alex R. Hains, Jonathan A. Allan, Luise Lago, Rebecca Sng

**Affiliations:** 1 University of Wollongong, Northfields Avenue, Wollongong, NSW 2522 Australia; 2 Illawarra Health and Medical Research Institute, Northfields Avenue, Wollongong, NSW 2522 Australia; 3 Illawarra Shoalhaven Suicide Prevention Collaborative, The Central, Innovation Campus, Wollongong, NSW 2500 Australia; 4 Coordinare, Primary Health Network South Eastern NSW, The Central, Innovation Campus, Squires Way, Wollongong, NSW 2500 Australia; 5 Centre for Health Research Illawarra Shoalhaven Population, University of Wollongong, Building 234, Innovation Campus, Wollongong, NSW 2500 Australia; 6 Grand Pacific Health, 336 Keira Street, Wollongong, NSW 2500 Australia

**Keywords:** Suicide prevention, Aftercare, Peer worker

## Abstract

**Background:**

Suicide is a major public health issue worldwide. Those who have made a recent suicide attempt are at high risk for dying by suicide in the future, particularly during the period immediately following departure from a hospital emergency department. As such the transition from hospital-based care to the community is an important area of focus in the attempt to reduce suicide rates. There is a need for evaluation studies to test the effectiveness of interventions directed to this stage (termed ‘aftercare’ interventions).

**Methods:**

A controlled non-randomised two group (intervention vs treatment-as-usual control) design, using an intention-to-treat model, will evaluate the effectiveness of a suicide prevention aftercare intervention providing follow-up after presentations to a hospital emergency department as a result of a suicide attempt or high risk for suicide. The intervention is a community-based service, utilising two meetings with a mental health clinician and follow-up contacts by peer workers via a combination of face-to-face and telephone for four weeks, with the option of extension to 12 weeks. Seventy-five participants of the intervention service will be recruited to the study and compared to 1265 treatment-as-usual controls. The primary hypotheses are that over 12 months, those who participate in the aftercare follow-up intervention are less likely than controls to present to a hospital emergency department for a repeat suicide attempt or because of high risk for suicide, will have fewer re-presentations during this period and will have lower all-cause mortality. As a secondary aim, the impact of the intervention on suicide risk factors for those who participate in the service will be evaluated using pre- and post-intervention repeated measures of depression, anxiety, stress, hopelessness, belongingness, burdensomeness, and psychological distress. Enrolments into the study commenced on 1 November 2017 and are anticipated to cease in November 2019.

**Discussion:**

The study aims to contribute to the understanding of effective interventions for individuals who have presented to a hospital emergency department as a result of a suicide attempt or at high risk for suicide and provide evidence in relation to interventions that incorporate peer-workers.

**Trial registration:**

ACTRN12618001701213. Registered on 16 October 2018. Retrospectively registered.

## Background

Suicide is a major public health issue worldwide [1]. In Australia, 3128 people died by suicide in 2017 (12.7 per 100,000), with suicide the leading cause of death amongst 15–44 year olds [2]. When it comes to suicidal behaviours more broadly, deaths are considered ‘the tip of the iceberg’ [3], with estimates of approximately 30 suicide attempts for every one suicide death [4]. Those who have made a recent suicide attempt are at high risk for dying by suicide in the future [5, 6], particularly immediately after discharge from a hospital emergency department [7] and within the first year following a suicide attempt [5, 8].

The most common point of contact that people who have attempted suicide have with support services is via a hospital emergency department [9]. However, people who have presented to an emergency department following a suicide attempt have described the support received as either insufficient [9] or completely absent [10–13]. As a result, there have been numerous calls to improve the support people receive within emergency departments and improve the way they are connected with ongoing community-based care [14, 15].

In the context of physical health care, the quality of the discharge planning and transfer of care is known to have significant impacts on patient outcomes [16–21]. Therefore, improved transition from hospital-based care to community supports for people presenting to an emergency department following a suicide attempt is a priority for effective suicide prevention [14, 15]. This type of intervention is referred to as ‘aftercare’.

Aftercare is consistently included as a key component of multilevel initiatives, or a ‘systems approach’ to suicide prevention [22–26]. One such systems approach is the *LifeSpan* project [23], which is being coordinated by the Black Dog Institute and implemented within four regions of New South Wales, Australia, including the Illawarra Shoalhaven region. The *LifeSpan* project involves implementing nine suicide prevention strategies simultaneously. Of all these strategies, implementing effective aftercare is estimated to reduce suicide attempts by 19.8%, the biggest impact from any one strategy in the *LifeSpan* systems approach [27].

The first landmark study evaluating the impact of a coordinated aftercare service was conducted on the outskirts of Oslo, Norway [28]. In what became known as ‘the Bærum Model’, people who presented to hospital after attempting suicide (but were not directly admitted) were followed up with home visits for up to 6 months and provided with support via a combination of face-to-face and phone contacts. There were four components to the support offered: (1) outreach – immediate, assertive outreach and follow-up after discharge from hospital, (2) problem-solving – solution focused counselling, (3) adherence – encouragement to remain engaged with treatment and follow recommendations, and (4) continuity – maintain contact with a consistent group of support people [29]. This intervention was credited with having contributed to a significant reduction in suicidal behaviours, from 170 per 100,000 in 1984 to 79 per 100,000 in 1995; a reduction of 53.5% [28].

These findings were supported by a Danish randomised controlled trial of a 6-month aftercare service based on the Bærum Model [30], in which 69 intervention participants were compared with 64 treatment-as-usual controls. The researchers found that significantly fewer people in the intervention group re-attempted suicide (8.7%) than the control group (21.9%) over a 12-month period. This study also found reduced numbers of repeated acts (8 in the intervention group versus 22 in the control group). The researchers noted that a key characteristic of the intervention was its “swift and rather aggressive outreach” [30] p.296, as well as the capacity to adapt the support to account for personal factors. A 5-year follow-up study found the number of people with repeated suicide attempts remained lower for the intervention group for up to 3–4 years, and the total number of repeat suicide attempts remained lower for the intervention group after 5 years [31].

Fundamentally, aftercare interventions aim to bridge the transition between an emergency department and community-based supports, with the key factor being a focus on making and keeping contact to ensure a constant sense of connectedness [31, 32]. As such, aftercare can be delivered via a number of methods – face-to-face contact [29], phone contact only [33, 34], a combination of phone and face-to-face [35], or mailed out letters [36–39]. The aftercare models evaluated have also ranged in duration, from a single contact [40] to regular contacts over 18 months [35]. Each of these models have illustrated the potential for aftercare services to have an impact on suicidal behaviours for those who have attempted suicide.

### Peer workers

Aftercare services have also varied in terms of their staffing profile, typically using a combination of doctors, nurses, psychologists, community workers, and social workers [41]. However, there are no records in the literature of aftercare services utilising *peer workers*; people employed on the basis of their personal lived experience of suicide or mental illness and recovery [42]. There is a growing body of evidence to support the use of peer workers in assisting people who are experiencing mental illness [42]. A review of four randomised controlled trials by Davidson, Chinman, Sells and Rowe [43] found that, across most outcomes, peer workers were just as effective as professionals in providing case management and support for individuals with severe mental illness. Furthermore, one of the studies reviewed found significantly fewer hospitalisations for those who were being case managed by peer workers compared to those being managed by a mental health professional [44]. Similarly, a reduction in the number of hospital re-admissions over a three-year period for mental health patients who received peer support has been observed [45]. These findings suggest there are potential benefits to involving peer workers in mental health care, although thus far, the majority of studies have been qualitative in nature and the few randomised controlled trials that have been conducted have primarily focussed on case management interventions [43, 46, 47]. A literature search conducted by the authors did not produce any studies that had specifically focussed on the use of peer workers with suicidal clients or in the context of aftercare services. As a result, there is a need for research to explore the effectiveness of a suicide prevention intervention utilising peer workers.

### GROW coaching model

In addition to providing a bridge between crisis (hospital-based) care and ongoing (community-based) care, the Bærum aftercare model also emphasised the importance of a practical problem-solving approach. An example of such an approach is provided by the GROW coaching model (Goal, Reality, Options, Wrap-up). The GROW model is a simple, collaborative, solution focussed framework that informs the structure of intervention sessions with clients [48–50]. Clients are firstly asked to set a goal (G) for what they would like to achieve during the coaching session. They are encouraged to create a goal that is specific and within their control, and they are assisted in breaking down larger goals into smaller, concrete steps [51]. Secondly, clients are assisted in gaining a better understanding of their reality (R), or current situation [49, 50]. They are encouraged to consider what they have previously found helpful, what they have found to be unhelpful, and any barriers they may be experiencing regarding their goals [51]. Thirdly, clients are encouraged to evaluate different options (O) for overcoming likely barriers to achieving goals [48, 51]. The fourth stage of the GROW model is the wrap-up (W) [49, 50], which encourages clients to create specific action steps in order to move forward, overcome barriers and evaluate their progress [48]. An advantage of a practical problem-solving framework, such as the GROW model is that it can be implemented, with relatively brief training, by staff of any background, including non-professionals.

### Targeting suicidal thoughts and behaviours

A critical factor in suicide prevention interventions is to directly target suicidal thoughts and behaviours [52]. An empirically supported example is the *Collaborative Assessment and Management of Suicide* (CAMS) model [53, 54], in which a clinician and client work collaboratively through goal-setting, treatment planning and monitoring [53]. Clients are asked to; outline the key problems or ‘drivers’ that are contributing to their suicidality and then work alongside a clinician to produce a set of goals and objectives to alleviate these problems; consider alternative behaviours to self-harm that they can engage in if in a suicidal crisis (e.g., going for a walk; calling an ambulance; people they can contact for help); and rate their suicide risk factors. Clients evaluate their goals and stabilisation plan at every treatment appointment [53, 54]. The structure of the CAMS framework is maintained within treatment sessions via the application of the *Suicide Status Form* (SSF), which is a tool that guides the conversation and collaborative work between the clinician and client.

Application of the CAMS framework has been shown to reduce suicidal ideation and/or behaviours amongst college students [54, 55] and defence personnel [56], as well as both outpatients [57, 58] and inpatients [59–61]. It has been found to be effective when adapted to online learning modalities [62] and has also demonstrated efficacy when delivered by clinicians from a range of disciplines [63]. The CAMS framework has not, to the authors’ knowledge, been applied within an aftercare context.

This paper describes the study protocol for a non-randomised controlled trial of an aftercare suicide prevention intervention that will provide follow-up to people who have presented to a hospital emergency department following a suicide attempt or with significant suicide risk, and who are not admitted to hospital as an inpatient, or being actively case managed by mental health services, at that time. The intervention, known in the region as ‘Aftercare’, will utilise clinicians and peer workers, and provide practical solution-focused intervention and targeted intervention of suicidal thoughts and behaviours. The Aftercare intervention will utilise the GROW coaching model and apply the SSF from the CAMS framework to target suicidal thoughts and behaviours directly. The intervention will involve a combination of face-to-face and telephone sessions over a four-week period, with the option of extension to 12 weeks.

## Methods/design

### Study aims

The primary objectives of the study are to evaluate the impact of the Aftercare intervention on the likelihood of a re-presentation to an emergency department as a result of a suicide attempt or because of high risk for suicide, the rates of re-presentation to emergency departments as a result of one or more repeat suicide attempt(s) or because of high risk for suicide, and the number of deaths by any cause. The secondary objectives of the study are to evaluate the impact of the Aftercare intervention on factors associated with increased risk of suicide, namely, mood [64], hopelessness [65, 66], belongingness, burdensomeness [66], and psychological distress [67, 68].

It is hypothesised that;

1. Participants of the Aftercare intervention will be less likely to have a repeat presentation to an emergency department within 12 months as a result of a suicide attempt or being at high risk for suicide than non-participants.
2. Participants of the Aftercare intervention will have lower rates of re-presentations to a hospital emergency department for suicide attempt or high risk for suicide within 12 months than non-participants.
3. Participation in the Aftercare intervention will be associated with fewer all-cause deaths within 12 months than non-participation.
4. For participants of the Aftercare intervention, depression, anxiety, stress, hopelessness, belongingness, burdensomeness, and psychological distress will be improved in comparison to baseline levels following the intervention.

### Study setting

The Aftercare intervention will be implemented in the Illawarra Shoalhaven region on the south-east coast of New South Wales (NSW), Australia. The region has a population of just over 400,000 and is served by three main public hospitals and several smaller regional hospitals [69]. The region experiences a suicide rate of 12.6 per 100,000, which is above the NSW state average of 10.6 per 100,000 [2], and more than 1000 suicide-related presentations to emergency departments each year [70].

The intervention will be implemented by a collaboration of three community-based organisations – Grand Pacific Health, Flourish Australia, and South Coast Medical Service Aboriginal Corporation. Referrals to the Aftercare service will be received by the service providers from the emergency departments of the three main hospitals.

### Study design

A controlled non-randomised two group (intervention vs treatment-as-usual (TAU) control) design will be used to test the primary hypotheses (hypotheses 1–3), following the intention-to-treat method. The secondary objectives of the study will be evaluated using a pre- and post-intervention repeated measures design (hypothesis 4), in which the intervention group (i.e. participants of the Aftercare intervention) will complete self-report questionnaires prior to their initial intervention session, and again at the time of their discharge appointment from the service.

#### Study duration

The Aftercare intervention commenced receiving referrals on 21 August 2017 from the emergency department of the largest hospital in the region, the Wollongong Hospital, and following the receipt of human research ethics approval, recruitment of study participants commenced on 1 November 2017. The service was rolled out gradually, so the full implementation of the intervention, including referrals from all three participating emergency departments, occurred on 5 March 2018. Data will continue to be collected for 12 months following the recruitment of the 75th intervention group study participant. As such, data collection for the study is forecast to cease by November 2020.

### Study sample

Participants in the Aftercare intervention will be drawn from people who attend one of the three participating emergency departments as a result of a suicide attempt or high risk for suicide, who meet the referral inclusion criteria. Participants’ risk for suicide (and therefore suitability for the Aftercare intervention) will be determined by the hospital staff using standard mental health assessment protocols. Those considered for referral to the Aftercare intervention will be 16 years of age and over. Those excluded from consideration for the service will be admitted to the hospital as an inpatient, or being actively case managed by mental health services, at that time of presentation to the emergency department. Those who consent to participate in the Aftercare intervention and who consent for their data to be included in the study will form the intervention group for the study.

The control group (TAU) will be sourced via de-identified hospital data extraction. They will be people 16 years of age and over who attend a participating emergency department as a result of a suicide attempt or high risk for suicide but either do not receive, or do not accept, a referral to the Aftercare intervention (i.e. did not wish to participate in the service; were unable to participate for other reasons e.g., time commitments; or had not received a referral to the service e.g. standard procedures were not followed in the emergency department). Pre-determined hospital codes for suicide attempt or high risk for suicide will identify controls. They will be selected by identifying those patients who presented to any of the emergency departments in the study during the study period, and identifying those who presented in the same month, who met the study inclusion criteria, and who had the closest emergency department presentation dates to the participants undertaking the Aftercare intervention and enrolled in the trial*.* A human research ethics waiver for consent was granted; all data is sourced via hospital data and code sets.

#### Inclusion and exclusion criteria

Health consumers who are aged 16 years of age and over who present to an emergency department with significant suicidality will be considered for the Aftercare intervention. They will be ineligible to participate if they are admitted to the hospital as an inpatient, if they are being actively case managed by the local health district’s mental health services, or if they have cognitive impairment sufficient to preclude full participation in, or comprehension of, the intervention as assessed by the referring clinician.

### Study procedure

**Fig. 1:**
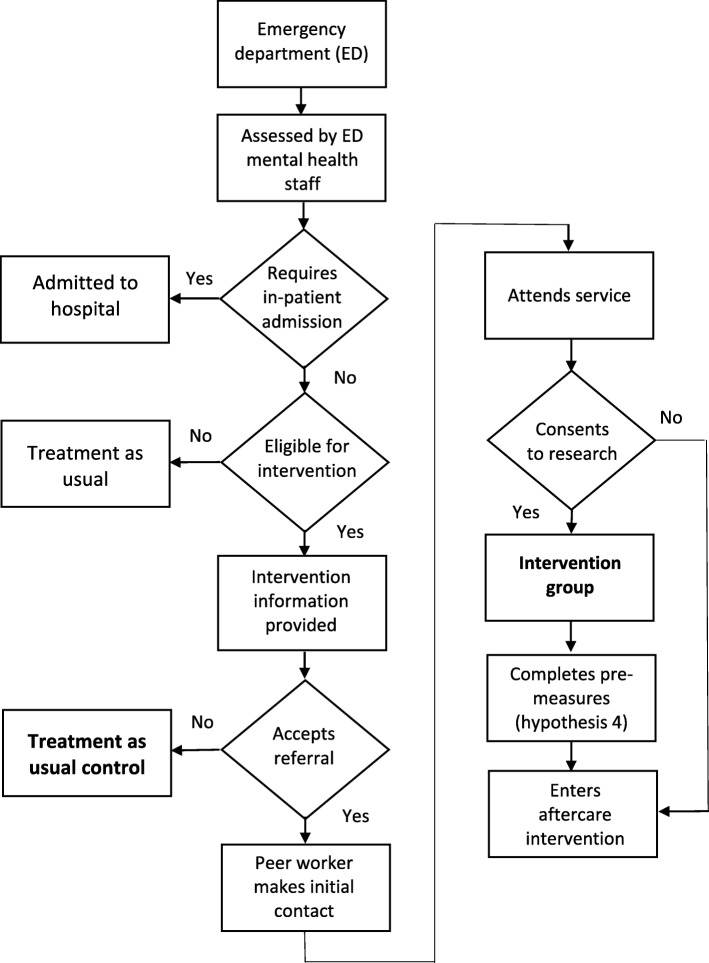
Participant Recruitment

Health consumers attending their local emergency department following a suicide attempt or at high risk for suicide are provided with a mental health assessment by clinical staff, where risk for suicide (and therefore suitability for the Aftercare intervention) is determined by standard hospital mental health assessment protocols. Staff determine whether the consumer warrants admission to hospital, as per the NSW Ministry of Health policies. If the consumer is not admitted to hospital as an inpatient, standard procedures include one follow-up phone call or home visit by the local health district’s mental health outreach team within seven days and a discharge letter to the person’s general practitioner. If the consumer is eligible for referral to the Aftercare service he/she will also be given a brief overview of the service by an emergency department clinician and at that time may choose to provide verbal consent to be referred to the service. The emergency department staff will contact the Aftercare service by phone and provide a referral. The participant flow process is shown in Fig. 1.

#### Aftercare intervention

The Aftercare service is administered through a collaboration of three community-based organisations in the region; Grand Pacific Health, Flourish Australia and the South Coast Medical Service Aboriginal Corporation. Referrals are taken by Grand Pacific Health and allocated according to location.

The consumer will be contacted by an Aftercare peer worker within one hour of the referral being received from the emergency department, while the consumer is still in the emergency department; or if the referral is made outside of operational hours they will be contacted the following day. A brief overview of the Aftercare program will be provided and, with consent, an initial community-based appointment with an Aftercare mental health clinician will be scheduled for the following day, or otherwise, as best convenient for the participant within the shortest possible timeframe.

Participants of the Aftercare service will attend the community-based service for their first appointment. Prior to their appointment, they will be provided with the research participant information sheet and consent form, and will have the opportunity to discuss the research further. Following this, they will complete the pre-intervention measures via tablet computer (or using pen and paper when appropriate).

The initial appointment with a mental health clinician will involve a collaborative assessment and planning session and take approximately one hour. The assigned peer worker will also be present and, with the consent of the participant, the participant’s family/carer will be encouraged to attend. Participants will complete the Suicide Status Form-4 (SSF-4) [53] with the mental health clinician. The SSF-4 is a multi-component clinical semi-structured assessment and treatment planning tool designed to assist in assessing the level of suicide risk and factors associated with suicidal thoughts. Six five-point Likert scales rate the degree of psychological pain, stress, agitation, self-hopelessness, self-hate, and overall suicide risk; and additional open-ended questions ask participants to elaborate upon these factors (e.g., “What I find most painful is…”). An earlier version of the form has demonstrated good convergent validity of these rating items with other established measures [55].

In addition, the SSF-4 has items which rate the degree to which participants attribute their suicidal thoughts and feelings to themselves (internal) or others (external), as well as their desire to live and their desire to die. Participants are also asked to make a list of their reasons for living versus their reasons for dying. The form then directs the interaction towards seeking solutions, via the question “the one thing that would make me no longer suicidal would be?” before the clinician gathers information regarding 14 risk factors for suicide (e.g. access to means, history of suicide attempts, substance use). The participant and clinician will document on the SSF-4 the agreed aftercare and stabilisation plans (e.g., “Things I can do to cope differently when I am in a suicide crisis”). The SSF-4 will be repeated at each contact with the clinician.

In the aftercare plan, participants will be asked to set personal goals, consider the values underpinning the goals, short-term and long-term action plans, their strengths and resources, and how to measure successful goal achievement. The aftercare plan, along with the stabilisation plan, will form the basis for individualised ongoing support, and will also be shared with the participant’s nominated general medical practitioner and other relevant stakeholders, with permission. Should family or other support people nominated by the participant attend the session, they will be provided with education on suicide risks and strategies to assist the participant to maintain progress toward his/her goals.

Peer workers will provide follow-up support sessions primarily via telephone (and several face-to-face) three times per week for four weeks; tapering to weekly should the intervention extend to 12 weeks. Each session will be approximately 30 min in duration. Participants will be coached through the steps in their aftercare plan using the framework of the GROW coaching model. Peer workers are trained in the SafeSide model [71] of risk formulation and will escalate to the mental health clinician should they be concerned about a participant’s level of risk.

The clinician and peer worker will meet for discussion and clinical review every two weeks, where they will consider the participant’s level of risk and progress, identify any barriers to progression and strategies to address these, and document any linkages to other services the peer worker has facilitated.

The mental health clinician will meet with the participant again at week four, together with the family/carer, if relevant. This face-to-face session will involve review of the aftercare and stabilisation plans, implementation of adjustments if indicated with a view to discharge from the service if appropriate. The participant and clinician will conduct the SSF-4 together and discuss any changes that may have occurred on the measures. Participants will be considered suitable for discharge when it is felt that their level of risk is appropriate relative to their level of support and the services with which they are engaged. If the participant is deemed suitable for discharge at this time, a discharge plan will be developed in collaboration with their peer worker and their family/carers. The participant and their family/carer (if relevant) will engage in relapse prevention planning and discharge letters will be sent to the participant’s general medical practitioner and any other relevant support services. Prior to discharge, the participant will complete the post-intervention outcome measures. If the participant is not deemed suitable for discharge at their week-four appointment with the clinician, the intervention may extend for up to a further eight weeks. Participants who receive 12 weeks of service and still present with elevated risk will be transitioned to other longer-term options, such as local health district mental health services.

#### Treatment as usual

Treatment as usual following a presentation to one of the participating emergency departments for a suicide-related event involves a mental health Clinical Nurse Consultant recommending, or actively referring, the patient to community-based supports (e.g. general practitioner, counselling, housing support services) and may include follow up by the community mental health team. However, there is no consistent support person to help coordinate these follow ups or check whether the person has in fact connected with any community-based supports. As per local policies, TAU includes one follow-up phone call or home visit by the local health district’s mental health outreach team within seven days. A discharge letter is also sent to the patient’s nominated general medical practitioner. Other intervention services may already be in place, or arranged as part of these interactions, as required.

### Staff training

#### Peer workers

Peer workers are drawn from an established peer worker organisation (Flourish Australia). They undergo a two-day training course in relation to the Aftercare intervention, which provides education regarding the SSF-4, working with aftercare and stabilisation plans, implementing the GROW coaching model, conducting risk assessments and using escalation procedures. Four peer workers are employed by the service.

#### Mental health clinicians

Aftercare mental health clinicians are registered practitioners with the Australian Health Practitioner Regulation Agency. Three clinical psychologists, two registered psychologists and one mental health nurse are employed by the service. All clinicians have attended a four-hour training course regarding the Aftercare intervention, focusing on the development of aftercare and stabilisation plans, the use of the SSF-4, implementation of the GROW coaching model, risk assessments, and procedures for the conduct of clinical review meetings with peer workers. In addition, clinicians have completed a comprehensive online suicide prevention training course, through the Australian Psychological Society, which provides information about suicide and suicidal behaviours, training in risk assessments and crisis management, training in working with populations at serious risk of suicide, information regarding appropriate referral options, and self-care for clinicians.

### Treatment Fidelity

Intervention fidelity will be assessed by measuring treatment adherence using participant’s attendance rates for peer worker follow-up and clinician appointments. To maximise intervention integrity, documented procedures will be in place within the Aftercare service to ensure that all workers comply with the intervention protocol. Measures of service indicators, such as adherence to meeting protocols and completion of SSF-4, will be made and audited to provide an assessment of intervention integrity.

### Measurements

#### Primary outcomes

Hospital emergency department data will be extracted to evaluate the primary outcomes of likelihood of a suicide-related presentation and rate of re-presentations to the three emergency departments as a result of one or more repeat suicide attempt(s) or being at high risk for suicide. Suicide attempt or high risk for suicide will be identified by pre-determined hospital codes. Death registration data will be linked with emergency department data for participants and controls to identify deaths by any cause.

#### Secondary outcomes

Secondary outcome measures were chosen as they assess factors associated with increased risk for suicide [64–68]. The measures will be collected from the participants of the Aftercare intervention (pre- and post-) using the following measures.

#### Hopelessness

The short form of the Beck Hopelessness Scale (BHS-SF) [72] is derived from the original Beck Hopelessness Scale [73] and consists of four True/False items designed to measure hopelessness in the past week. An example of an item is “I might as well give up because I can’t make things better for myself”. The BHS-SF has demonstrated high internal consistency (α = .85) and has been found to be positively correlated with dysfunctional attitudes, exhaustion, psychological distress, hostility, lack of life goals and inability to cope emotionally [72].

#### Belongingness and burdensomeness

The 15-item Interpersonal Needs Questionnaire (INQ) utilises a seven-point Likert Scale (1 = not at all true for me, 7 = very true for me) designed to measure thwarted belongingness and perceived burdensomeness [74]. An example of an item measuring thwarted belongingness is “These days I am fortunate to have many caring and supportive friends” (reverse scored). An example of an item measuring burdensomeness is “I think my death would be a relief to the people in my life”. Both the thwarted belongingness and perceived burdensomeness scales have shown good internal consistency (α = .85 and α = .89, respectively) and have been found to demonstrate criterion validity [74, 75].

#### Depression, anxiety and stress

The 21-item version of the Depression, Anxiety and Stress Scales (DASS-21) [76] measure items on a four-point Likert Scale (0 = did not apply to me at all, 3 = applied to me very much or most of the time) designed to gauge symptoms of depression, anxiety and stress. The DASS-21 has an acceptable level of internal consistency with Cronbach’s alphas for the three scales ranging from .73 (anxiety) to .81 (depression and stress) [77]. The DASS-21 has been well validated [78–80]. The depression scale of the DASS-21 has been found to predict suicidal ideation [81].

#### Psychological distress

The 10-item Kessler Psychological Distress Scale (K-10) [82] consists of 10 items rated on a five-point scale Likert Scale (1 = none of the time, 5 = all of the time) designed to measure psychological distress over the past four weeks, e.g., “About how often did you feel so nervous that nothing could calm you down?” The K-10 has demonstrated good reliability and validity [83].

### Data handling

Primary outcome data of re-presentations to an emergency department and other local health district data on hospital admissions, community mental health and mental health outcomes will be extracted and de-identified by the Centre for Health Research Illawarra Shoalhaven Population (CHRISP), a research centre which performs data linkage through a partnership between the local health district and the University of Wollongong. Deaths attributable to any cause will be obtained from NSW death registration data held by the NSW Centre for Health Record Linkage (CHeReL). The researchers with be provided with the de-identified coded data.

Secondary outcome data (suicide risk factors) will be collected by the Aftercare service prior to the participant attending their initial session with a mental health clinician and again at the time of their final (discharge) session with the clinician. For Aftercare participants who consent for their data to be included in the study, the data will be de-identified and coded within the Aftercare service, prior to being provided to the researchers.

Data transfers and storage will comply with national and state legislation, national ethics principles and University of Wollongong, local health district and the CHRISP data management policies.

#### Data linkage

The CHRISP data integration officer will link Aftercare and local health district identifiers using deterministic linkage on the Illawarra Health Information Platform. CHeReL will link CHRISP’s patient identifier with their own patient identifier using deterministic linkage in the first instance, and probabilistic linkage if required. Probabilistic record linkage allows a calculation of the probability of a match between two or more records to be made when the records are not exactly identical in one or more of the participant identifier fields (e.g., surname ‘Browne’ or ‘Brown’). A record linkage is determined by set thresholds for acceptable probability [84]. Non-identifiable local health district data will be provided to the researchers by CHRISP. Non-identifiable Aftercare service data will be provided to the researchers by the Aftercare service. Non-identifiable deaths data from the state’s Registry of Births, Deaths and Marriages will be provided to the researchers by CHeReL.

### Data analysis

Analyses will be conducted using the Statistical Package for the Social Sciences version 25 [85] and SAS v9.4 [86]. Primary analyses will be conducted according to the intention-to-treat approach. Presentations to an emergency department with a repeat suicide-related event and for all-cause death will be compared between the two non-randomised groups (intervention vs control) using a Kaplan Meier plot where the initial presentation to an emergency department is designated as time zero, and using censoring. A log-rank test will compare the Kaplan Meier curves of the two groups.

A negative binomial regression model will be applied to the data to estimate the effect of the intervention on re-presentation rates, accounting for varying lengths of follow-up period and adjusting for service and person-level characteristics (e.g. facility, age, sex, indigenous status) and censoring.

To investigate the impact of the service on suicide risk factors (secondary outcome measures), a comparison of pre- and post-intervention outcome measures will be conducted for participants of the intervention using paired t-tests, or a Wilcoxon signed-ranks test (if the sample is non-normally distributed), adjusting for multiple comparisons (Bonferroni correction).

### Sample size and statistical power

The sample size required to achieve power of 80% was estimated for the primary analyses using G*Power [87]. Local hospital data (2014/2015 year) indicated that approximately 1340 people present to emergency departments with suicide-related presentations annually and systematic reviews have estimated that 16% re-attempt suicide within 12 months [5]. Based on Hvid et al.’s [30] report that an intervention group had approximately half the rate of deaths by suicide than a TAU group at 12 months (.087 vs .219), the sample size required for a log-rank test at α level .05 is 75 participants in the intervention group and 1265 participants in the control group.

## Discussion

This study aims to contribute to our understanding of effective interventions for people at high-risk for suicide, with the intention of quantifying any impact on the number of presentations to emergency departments for suicide-related events, and all-cause mortality, following an aftercare intervention. The study will provide a real-world examination of an innovative aftercare follow-up service for people who have presented to a hospital emergency department following a suicide attempt or because of high risk for suicide. Specifically, the study will expand upon our understanding of follow up in suicide prevention [35, 88], extend literature regarding peer worker integration in treatment models of suicidal persons, and inform future policy decisions.

### Limitations

The study groups are non-randomised, hence intervention participants are self-selecting and may therefore constitute a group that is inherently different from controls prior to study commencement, such as having higher motivation to seek treatment or greater levels of psychological mindedness. Controlling such factors will require future randomised controlled trials. The current study is typical of pragmatic trials which test the effectiveness of interventions in a real-world context as opposed to efficacy trials which are in controlled conditions.

## Data Availability

The datasets generated and analysed during the current study are not publicly available due to privacy concerns but are available from the corresponding author on reasonable request and with permission of Grand Pacific Health and the Centre for Health Research Illawarra Shoalhaven Population.

## Abbreviations

BHS-SF: Beck Hopelessness Scale
CAMS: Collaborative Assessment and Management of Suicide
DASS-21: Depression, Anxiety and Stress Scales
GROW: ‘Goal, Reality, Options, Wrap-up’ coaching model
INQ: Interpersonal Needs Questionnaire
K-10: Kessler Psychological Distress Scale
NSW: New South Wales
SSF: Suicide Status Form
TAU: treatment-as-usual
